# Transient Stability of Epigenetic Population Differentiation in a Clonal Invader

**DOI:** 10.3389/fpls.2018.01851

**Published:** 2019-03-01

**Authors:** Wen Shi, Xiaojie Chen, Lexuan Gao, Cheng-Yuan Xu, Xiaokun Ou, Oliver Bossdorf, Ji Yang, Yupeng Geng

**Affiliations:** ^1^Yunnan Key Laboratory for Plateau Mountain Ecology and Restoration of Degraded Environments, Institute of Ecology and Geobotany, School of Ecology and Environmental Science, Yunnan University, Kunming, China; ^2^Biocontrol Engineering Research Center of Plant Disease & Pest, School of Life Sciences, Yunnan University, Kunming, China; ^3^Biocontrol Engineering Research Center of Crop Disease & Pest, School of Life Sciences, Yunnan University, Kunming, China; ^4^Key Laboratory for Biodiversity Science and Ecological Engineering, Ministry of Education, Fudan University, Shanghai, China; ^5^School of Health, Medical and Applied Sciences, Central Queensland University, Rockhampton, QLD, Australia; ^6^Plant Evolutionary Ecology, Institute of Evolution and Ecology, University of Tübingen, Tübingen, Germany

**Keywords:** alligator weed, *Alternanthera philoxeroides*, biological invasions, clonal plants, DNA methylation, epigenetic variation, phenotypic plasticity, population differentiation

## Abstract

Epigenetic variation may play an important role in how plants cope with novel environments. While significant epigenetic differences among plants from contrasting habitats have often been observed in the field, the stability of these differences remains little understood. Here, we combined field monitoring with a multi-generation common garden approach to study the dynamics of DNA methylation variation in invasive Chinese populations of the clonal alligator weed (*Alternanthera philoxeroides*). Using AFLP and MSAP markers, we found little variation in DNA sequence but substantial epigenetic population differentiation. In the field, these differences remained stable across multiple years, whereas in a common environment they were maintained at first but then progressively eroded. However, some epigenetic differentiation remained even after 10 asexual generations. Our data indicate that epigenetic variation in alligator weed most likely results from a combination of environmental induction and spontaneous epimutation, and that much of it is neither rapidly reversible (phenotypic plasticity) nor long-term stable, but instead displays an intermediate level of stability. Such transient epigenetic stability could be a beneficial mechanism in novel and heterogeneous environments, particularly in a genetically impoverished invader.

## Introduction

Epigenetic modifications can modulate gene expression without altering the underlying DNA sequence (Jones, 2012). Recently, the study of epigenetic modifications, i.e., epigenetics, has attracted increasing attention of ecologists and evolutionary biologists because epigenetic processes may play a role also in the ecology and evolution of natural populations. Specifically, epigenetic variation among individuals can be a source of phenotypic variance within and among plant populations (Herrera and Bazaga, 2011; Medrano et al., 2014; Kooke et al., 2015; Zhang et al., 2018) and can affect their ecological performance, niche breadth, evolutionary potential and invasion success (Bossdorf et al., 2010; Richards et al., 2010; Herrera et al., 2012; Latzel et al., 2013; Zhang et al., 2013; Hawes et al., 2018). In addition, epigenetic modifications are important for genomic stability during plant hybridization and polyploidization, thus paving the way for genome evolution (Bossdorf et al., 2008; Richards, 2008). These initial observations stimulated a new discipline of ecological epigenetics (Bossdorf et al., 2008; Richards et al., 2017) which focuses on the causes and consequences of epigenetic variation in natural populations. Currently, much of our knowledge on plant epigenetics is from model species like *Arabidopsis thaliana* (Schmitz et al., 2013; Heard and Martienssen, 2014). We know that epigenetic modifications can occur spontaneously or plastically in response to environment stimuli (Richards et al., 2017), that many epigenetic modifications are reset during mitosis or meiosis, but that others are stably maintained throughout the life time of organism, or even transmitted across generations (Richards, 2008; Richards et al., 2010). Such stable epigenetic modifications may provide additional raw material for natural selection to act upon (Bossdorf et al., 2008). For understanding the ecological significance of epigenetics, however, it is important to test whether findings in *Arabidopsis* also hold for wild species, and to what extent natural epigenetic variation is stable enough to play a role in the evolution of plant populations under field conditions (Kalisz and Purugganan, 2004; Richards, 2008; Richards et al., 2017).

The currently most studied epigenetic modification in ecological epigenetics is DNA methylation. It can be investigated easily in large numbers of individuals sampled from natural populations using Methylation Sensitive Amplified Polymorphism (MSAP) markers (Angers et al., 2010), a modification of the AFLP technique. In the past years, MSAP studies often found significant epigenetic population differentiation in wild plant populations, and that epigenetic variation is associated with environment (see Kilvitis et al., 2014 for a recent review). These patterns were confirmed in different plant species (Herrera and Bazaga, 2010; Lira-Medeiros et al., 2010; Richards et al., 2012). However, the origins and stability of epigenetic-environment association often remained unclear. One possibility is that epigenetic differences observed in natural populations are environmentally induced and are therefore reversible when environments change, i.e., they reflect phenotypic plasticity. Another possibility is that these epigenetic differences result from spontaneous epimutation, have been shaped by natural selection and/or (epi-)genetic drift, and are stable across generations (Richards et al., 2017). To test these two contrasting hypotheses, it is necessary to analyze the dynamics of epigenetic population variation both in the field and under common environmental conditions (Bossdorf et al., 2008). Since environmentally induced epigenetic variation could also have a transient stability, i.e., persist across a limited number of generations, a multi-generation common-garden approach is particularly powerful (Whipple and Holeski, 2016).

An important question in ecological epigenetics is how important epigenetic variation is relative to genetic variation. Although many epigenetic modifications may be partly or completely controlled by DNA sequence (“obligatory” or “facilitated” epigenetic variation *sensu* Richards, 2008), others may be independent of DNA sequence (“pure” epigenetic variation). From an evolutionary perspective, pure epigenetic variation is especially interesting because, if heritable and related to phenotype, it provides additional phenotypic variation and thus broadens the potential for evolution and adaptation, even in species lacking DNA sequence variation (Bossdorf et al., 2008). However, the complex interactions between genetic and epigenetic processes make it very difficult to evaluate these questions in natural populations of many species, in which the two factors are often confounded (Bossdorf et al., 2008). One solution to isolate epigenetic processes for more thorough study is to use asexual organisms as research system. In plants, asexual reproduction is widespread, so individuals occurring in different habitats may belong to the same clone lineage, thus providing natural replication of nearly identical genomes across contrasting environments. Moreover, epigenetic variation within the same lineage is necessarily independent of DNA sequence, thus providing opportunities for studying epigenetics-environment relationships in natural populations without the confounding effects of genetic variation (Bossdorf et al., 2008; Richards, 2008; Douhovnikoff and Dodd, 2015).

Some researchers have argued that epigenetic processes may be particularly relevant for the ecological success of asexually reproducing species, because they may generate phenotypic variation even in genetically uniform clonal stands, and thus allow these species to acclimate or adapt to new environments (Castonguay and Angers, 2012; Verhoeven and Preite, 2014; Douhovnikoff and Dodd, 2015). In clonal plants that continuously produce new modules, epigenetic modifications could accumulation over time and result in progressive acclimation (Douhovnikoff and Dodd, 2015).

Some asexually reproducing plants are highly successful invasive species that occur across broad geographic and environmental ranges (Pyšek, 1997; Silvertown, 2008). One of the most dramatic examples is alligator weed (*Alternanthera philoxeroides*), a native to South America which has become invasive in many countries (Holm et al., 1997). Alligator weed can form dense monocultures through clonal growth and cause substantial ecological and economic damage (Li and Xie, 2002). In the native range of alligator weed, both sexual and asexual reproduction are observed (Sosa et al., 2007), but invasive Chinese alligator weed populations are dominated by asexual reproduction, and DNA marker studies found them to be genetically uniform (Xu et al., 2003; Ye et al., 2003). Despite this lack of genetic variation, alligator weed occurs across a broad geographic and climatic range and in highly heterogeneous habitats in China (Pan et al., 2006; Chen et al., 2008; Geng et al., 2016). Previous studies showed that morphological plasticity and clonal integration may contribute to the adjustment of alligator weed to heterogeneous habitats on small spatial scale (e.g., terrestrial vs. aquatic, Geng et al., 2007; Wang et al., 2009; Gao et al., 2010; Xu et al., 2010; You et al., 2014). However, the mechanisms underlying the species’ adjustment to large-scale environmental variation, such as climate differences, are not clear yet. In a previous study, we found significant epigenetic differentiation not only among different habitat types but also among three geographically distinct populations (Gao et al., 2010), suggesting a potential role of epigenetic processes at larger scales. However, the origin and stability of these epigenetic differences are still unknown.

Here, we studied epigenetic variation in genetically uniform invasive Chinese populations of alligator weed. We repeatedly analyzed DNA methylation in populations from different climatic areas, as well as in multiple generations of their offspring grown in a common environment. This allowed us to assess the stability and consistency of epigenetic population differentiation, taking advantage of alligator weed as an excellent model system for studying pure epigenetic variation.

## Materials and Methods

### Study Species

**FIGURE 1:**
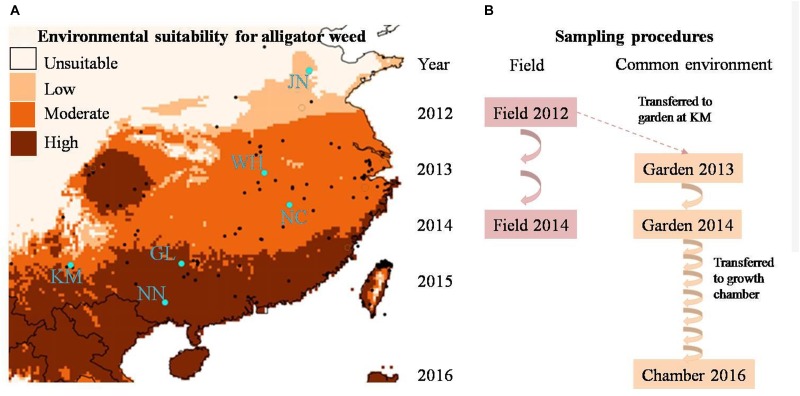
**(A)** The geographic distribution of alligator weed field sites (blue dots) and **(B)** multi-generation experimental design of our study. In **(B)** the arrows indicate the numbers of generations in the field and common environment, respectively.

Alligator weed (*Alternanthera philoxeroides*) is a stoloniferous, perennial herb native to South America. In China, the earliest herbarium specimens are from Shanghai in the 1930s, and from other areas of Eastern China in the 1940s (Chen et al., 2008). During the 1950–1970s, the geographic distribution of alligator weed rapidly increased because it was introduced as a fodder crop to many provinces, where it subsequently escaped and established wild populations (Chen et al., 2008). Thus, most Chinese populations have a short history of less than 70 years. At present, alligator weed occupies a geographic range from 20 to 40 degree northern latitude (Figure 1A), covering a broad climatic range from tropical to sub-tropical and temperate climate. Alligator weed produces small clover-like white flowers in the summer, but the flowers usually drop before the seeds are mature. Alligator weed has a very vigorous asexual reproduction, with small stem or rhizome fragments rapidly developing into new individuals (Dong et al., 2012; Guo and Hu, 2012). In Northern China, all aboveground biomass of alligator weed dies during the cold season, but belowground roots and rhizomes remain alive and can re-sprout in the spring. In southern areas with mild winter, in contrast, alligator weed grows throughout the year. Asexual reproduction greatly contributes to the species’ rapid spread (Guo and Hu, 2012). In aquatic habitats, broken stem fragments can disperse long-distance by water flow, and in terrestrial habitats rhizome fragments of alligator weed are often spread unintentionally over long distance as soil contaminants. Thus, the population regeneration and spread of alligator weed in China is entirely by asexual means. Indeed, molecular marker analyses showed that many invasive populations across large climatic gradients belong to the same clone (Xu et al., 2003; Ye et al., 2003), which make the species an intriguing study system for *in situ* ecological epigenetic studies.

### Field Sampling

To encompass the climate range experienced by alligator weed in China, we selected six invasive Chinese populations, ranging from N°22.8 to N°36.5 in latitude (Figure 1A), and all from terrestrial habitats. In China, alligator weed occurs in a variety of habitats including aquatic and terrestrial ones (Geng et al., 2007), but in this study we wanted to focus on large-scale variation of alligator weed driven by climate rather than smaller-scale habitat variation, and we therefore collected samples only from terrestrial habitats that are more strongly affected by climate than aquatic ones. In summer 2012, we sampled 10 healthy ramets from each population. To ensure that the ramets were not from the same physiological individual, we kept a minimum distance of 10 m between samples. Moreover, to minimize epigenetic variation caused by differences in plant development, we always sampled the fourth or fifth pair of mature leaves from the stem apex. All leaf samples were dried and stored in plastic bags with silica gel until their use for genetic and epigenetic analyses. In addition to the leaf samples, we also collected the stems (apex to sixth or seventh node) of the same ramets for setting up the common garden experiment (see below). To be able to assess the temporal stability of epigenetic variation in the field, we repeated the leaf sampling again in summer 2014 in the same populations, using the same protocol as in 2012.

### Common-Environment Experiments

To assess the heritability of epigenetic variation observed in the field, and thereby disentangle plasticity from heritable variation, we set up a common-garden experiment at Yunnan University in Kunming. All 60 ramets collected from the six invasive populations were planted individually into 4-L plastic pots filled with a 1:1 mixture of vermiculite and sand and placed randomly in an outdoor garden where they were exposed to ambient temperature and precipitation. Each pot was fertilized with 4 g of slow-release fertilizer (N:P:K ratio of 15:11:13; Osmocote controlled release all purpose fertilizer, the Scotts Company, Marysville, OH, United States) and was watered regularly with tap water. The common garden experiment ran for 2 years. After 12 months, we collected leaf material for epigenetic analyses, using the same protocol as in the field sampling. Thereafter we removed all remaining leaves and shoots, leaving only a stem segment with six nodes which was then re-planted into a pot with fresh substrate. The plants regenerating from these stems were considered the second asexual generation. We repeated the procedure in summer 2014, resulting in a third asexual generation (Figure 1B).

Since our molecular analyses of the first asexual generations showed a gradual decrease, but not complete loss, of epigenetic population differentiation (see below), we were asking ourselves whether epigenetic differences between populations would eventually completely disappear. Therefore, to fast-forward the asexual generation cycle, we moved the plants to a growth chamber (Percival^®^ E-36L2) in April 2016, where the plants experienced constant benign growth conditions, with a 12/12 h day: night cycle at 30°C/25°C, and a much faster generation time of one asexual generation per month. Because of space limitation, only half of the common-garden plants (5 randomly selected individuals per population) could be moved to the growth chamber. In October 2016, after six additional asexual generations in the growth chamber, we collected a final batch of samples from the altogether tenth asexual generation, for molecular analyses, using the same protocol as in the field and common garden. Altogether, we obtained plant samples from two time points in the field (2012 and 2014) and from three time points in a common environment (2013 and 2014 from the common garden and 2016 from the growth chamber, Figure 1B) to study the stability of epigenetic population differentiation.

### Molecular Lab Work

We isolated total genomic DNA from all silica gel-dried leaf samples using the TIANGAN Plant Genomic DNA kit (Tiangen Biotech, Beijing, China) following the standard manufacturer protocol. The DNA samples were then dissolved in 50 μl TE buffer and stored at -20°C. To assess the genetic variation within and among invasive populations we used amplified fragment length polymorphism (AFLP) fingerprinting, following the standard protocol Vos et al. (1995) with some modifications (Gao et al., 2010). We used nine EcoRI/MseI primer combinations for selective amplification: AGG/CAA, AGC/CAA, AAC/CTT, ACA/CTA, CAA/CAT, AGC/CTT, AGC/CTA, AGG/CTT, and AGG/CAT. The epigenetic variation among and within populations was analyzed with the methylation-sensitive amplified polymorphism (MSAP) technique, which is related to AFLP markers and follows the same protocol as described above, except that the frequent cutter MseI was replaced by methylation-sensitive restriction enzymes HpaII and MspI. HpaII and MspI are a pair of isoschizomers which can both cleave 5′-CCGG sequences but have different sensitivities to the methylation at internal or external cytosine (Schulz et al., 2013). The differences in the final PCR products thus reflect different methylation states at the cytosines of CCGG sites and allow detecting epigenetic differences among plant samples. We conducted MSAP analyses of all alligator weed samples using ten EcoRI+ HpaII/MspI primer combinations each with three selective nucleotides: AAG/TCC, ACA/TCG, ACT/TCT, ACC/TGA, AGA/TTC, AGG/TTG, AAC/TCT, AAG/TTC, AAC/TGA, and AAC/TCAA. The fragments were separated on 6% sequencing gels and silver-stained as described above and scanned for band scoring. To assess the reproducibility of our analyses, we repeated the half of the MSAP analyses with independent DNA isolations. Throughout the molecular analyses, all samples were randomized to avoid any systematic biases or errors.

### Data Scoring

To obtain multilocus genotypes and epigenotypes for all plants, we scored all reproducible fragments between 100 and 500 bp as present (1) or absent (0) for AFLP and MSAP data, generally excluding samples of poor visual quality. All fragment scoring was done by the same person unaware of sample identities. The AFLP data was scored as a binary matrix following the standard protocol (Gao et al., 2010), whereas the status of MSAP loci was determined through comparison of the EcoRI/HapII and EcoRI/MspI fragment profiles, with four possible outcomes: (I) fragments were present in both profiles (1/1), (II) fragments were present only with EcoRI/MspI (0/1), (III) fragments were present only with EcoRI/HpaII (1/0), or (IV) fragments were absent with both cutters (0/0). The first three outcomes indicate different methylation status, while the last outcome is uninformative because it can have different causes including methylation variation or DNA sequence mutation (Schulz et al., 2013). Since in our study alligator weed harbored hardly any genetic variation, which was consistent with previous findings (Xu et al., 2003), we considered the fragments of type IV (0/0) as methylated and included them in our dataset. The raw MSAP data was thus a multi-state matrix containing condition I, II, III and IV. Before further analyses, we transformed this matrix into a binary matrix following the ’Mixed Scoring 2′ method of Schulz et al. (2013), which distinguishes between three types of markers: m-type (full methylation), h-type (hemimethylation) and u-type (no methylation). Monomorphic loci were generally excluded from the data set to avoid biases in parameter estimation (Bonin et al., 2004).

### Data Analyses

We analyzed the binary AFLP and MSAP data sets with a band-based strategy (Bonin et al., 2004) and used the R script by Schulz et al. (2013) to calculate genetic and epigenetic diversity within populations, as well as the percentage of polymorphic loci and Shannon’s diversity index. Due to the extremely low levels of genetic diversity revealed by the AFLP markers, all further analyses were done only for the MSAP data. First, we visualized patterns of epigenetic variation through principal coordinate analyses (PCoA) based on a matrix of Nei and Li distances calculated with DISTAFLP (Mougel et al., 2002). The distance matrices were square root-transformed to meet the assumptions of PCoA (Legendre and Legendre, 1998). Second, we calculated a hierarchical AMOVA to test for the significance of epigenetic differentiation among populations and groups (five different growth environments and/or years of sampling), with the probability of non-differentiation (PhiPT = 0) estimated over 9,999 permutations. In addition, we also calculated pairwise PhiPT comparisons (an analog of the *F*_ST_ index) between populations within each of the five groups, plus pairwise comparisons of different groups using Kruskal–Wallis rank sum tests. Last, we ran Mantel tests to test for relationships between AFLP and MSAP distances of individuals, and between genetic, epigenetic and geographic distances at the population level. PCoA, AMOVA, and Mantel test were done with GenAlex 6.5 (Peakall and Smouse, 2012), the Kruskal–Wallis rank sum test in R.

To better understand the dynamics of DNA methylation across generations, we further analyzed the stability of individual epiloci (i.e., conditions I, II, III, and IV) following the method of Herrera et al. (2013), where the stability of an epilocus is defined as the proportion of plants with unchanged DNA methylation status across time, in our case experimental generations. From our technical controls we knew that the error rate of MSAP markers was 1.71%, so we considered loci with stability above 98.29% as ‘stable.’ We tested the stability of epiloci from the field to the common environment was estimated for three different durations: (1) across two generations, the 2012 field data and 2013 garden data, (2) across three generations, the 2012 field data and both 2013 and 2014 garden data, and (3) across 10 generations, the 2012 field data, 2013 and 2014 garden data and 2016 growth chamber data.

## Results

### Genetic and Epigenetic Diversity in the Field

We scored a total of 469 AFLP bands and found only six polymorphic AFLP loci (1.28%) and five distinct multi-locus genotypes. One dominant genotype represented 44 of the 60 samples (73.3%) and occurred in all six populations along the geographic gradient. At the population level, the average percentage of polymorphic loci was 0.34% and the average Shannon’s diversity was 0.002, indicating extremely low genetic diversity (Table 1). There was no significant genetic differentiation among populations (PhiPT = 0.036, *P* = 0.171) and no isolation-by-distance (*r* = 0.227, *P* = 0.202) at the genetic level.

**Table 1:** Genetic and epigenetic diversity of six populations of *Alternantheraphiloxeroides*in the field, common garden, and growth chamber.

	GL	JN	KM	NC	NN	WH	Mean
**%Polymorphic loci**		
AFLP loci (469)							
Field 2012	0.42	0.21	0.21	0.83	0.21	0.21	**0.34**
MSAP sub-loci (732)							
Field 2012	9.29	2.60	6.28	7.24	7.79	4.92	**6.35**
Field 2014	8.61	5.74	7.38	7.51	10.93	4.78	**7.49**
Garden 2013	8.88	1.50	4.92	6.01	8.20	7.92	**6.24**
Garden 2014	7.51	2.05	6.97	7.51	7.51	6.15	**6.28**
Chamber 2016	1.23	0.96	1.23	1.09	3.42	1.37	**1.55**
**Shannon’s diversity**		
AFLP loci (469)							
Field 2012	0.003	0.001	0.002	0.002	0.005	0.001	**0.002**
MSAP sub-loci (732)							
Field 2012	0.067	0.017	0.042	0.047	0.057	0.034	**0.044**
Field 2014	0.055	0.036	0.046	0.043	0.079	0.033	**0.049**
Garden 2013	0.064	0.009	0.035	0.039	0.057	0.055	**0.043**
Garden 2014	0.063	0.016	0.057	0.061	0.064	0.051	**0.052**
Chamber 2016	0.011	0.008	0.01	0.01	0.028	0.011	**0.013**


**FIGURE 2:**
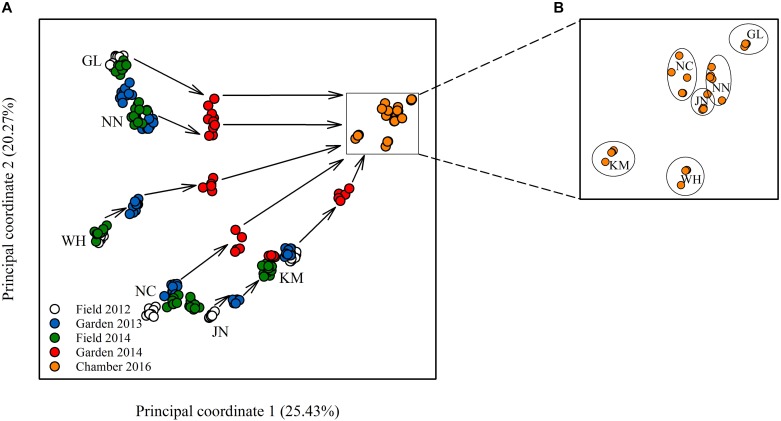
Plot of principal coordinate analysis showing **(A)** the relative epigenetic distances among the different populations in the field and common environments, and, in a zoom-in, **(B)** the population clustering remaining after 10 asexual generations.

Epigenetic diversity, in contrast, was much higher within and among populations in the field. Out of a total of 510 MSAP multi-state markers (i.e., four possible outcomes with two restriction enzymes), 369 were polymorphic (77.65%). When we re-coded the MSAP multi-state markers into binary data (following Schulz et al., 2013), this resulted in 732 polymorphic subloci (m-, h-, and u-type). At the population level, epigenetic diversity was nearly 20-fold higher than genetic diversity, with an average percentage of polymorphic loci of 6.35% and an average Shannon’s diversity of 0.044 (Table 1). When these analyses were done separately for the three types of sub-loci, the patterns were similar (Supplementary Table S1). AMOVA indicated significant epigenetic differentiation among populations (PhiPT = 0.894, *P* < 0.001) (Table 2), and principal coordinates analysis (PCoA) also showed a clear separation of the six populations, based on their epigenetic variation in the field in 2012 (Figure 2). Mantel tests showed that epigenetic variation (at the level of individuals) was independent of genetic variation (*r* = 0.02, *P* = 0.272), and that there was (at the population level) a non-significant negative correlation between epigenetic and geographic distance (*r* = -0.287, *P* = 0.171).

**Table 2:** Results of AMOVA of six populations of *Alternanthera philoxeroides* in the field, for AFLP data and for MSAP data from different years and growth environments.

	Variance among populations	Variance within populations	*P*-value	Phi-statistics
**AMOVA results for AFLP data**				
Field 2012	0.009 (4%)	0.248 (96%)	0.171	0.036
**AMOVA results for MSAP data**				
Field 2012	67.438 (89%)	7.957 (11%)	0.000	0.894
Field 2014	58.120 (87%)	8.724 (13%)	0.000	0.869
Garden 2014	55.817 (88%)	7.819 (12%)	0.000	0.877
Garden 2014	39.011 (78%)	11.200 (22%)	0.000	0.777
Chamber 2016	36.861 (93%)	2.783 (7%)	0.000	0.930


### Temporal Stability of Epigenetic Variation

Comparison of the epigenetic profiles of the 2 years of field samples showed that the epigenetic population differentiation observed under field conditions was remarkably stable across years. Not only were the levels of epigenetic diversity (Table 1) and the overall level of population differentiation (Table 2) similar, but the 2012 and 2014 samples from the same populations generally occupied very similar positions in the PCoA space (Figure 2). In the common environment, in contrast, the epigenetic variance among populations gradually decreased (Table 2), and populations became more similar with increasing numbers of asexual generations (Field 2012 to Garden 2013, Garden 2014 and Chamber 2016 in Figure 2). However, even in the growth chamber in 2016, after 10 asexual generations, there was still significant epigenetic population differentiation (Table 2). These results were confirmed by the stability analyses of individual epiloci, where around half of the epiloci remained unchanged between two successive generations, but the proportion of changed epiloci increased significantly with increasing numbers of generations (Table 3). Nevertheless, even after 10 asexual generations, there were still 38% of the epiloci unchanged. The stability of epiloci also depended on its methylation status: the most stable epiloci were fully methylated ones (type I; 1/1) whereas unmethylated ones (type IV; 0/0) were the least stable. Interestingly, the % epigenetic variance residing among populations did not change much across the different times points in the common environment, indicating that epigenetic differences among and within populations must have decreased at similar rates. Likewise, the overall levels of epigenetic diversity did not change from 2012 in the field to 2013 and 2014 in the common garden, and they only dropped strongly when the plants were grown in the much more homogenous environment of the growth chamber (Table 1).

**Table 3:** Stability of *Alternantheraphiloxeroides* epiloci across different numbers of generations, and the fractions of stable epiloci residing in the different epiloci types.

	Field 2012 to Garden 2013	Garden 2013 to Garden 2014	Field 2012 to Garden 2014	Field 2012 to Chamber 2016
# Changed epiloci	232 (45.5%)	202 (39.6%)	288 (56.5%)	315 (61.76%)
# Stableepiloci	278 (54.5%)	308 (60.4%)	222 (43.5%)	195 (38.24%)
**Types ofstableepiloci**		
Type I (1/1)	58.80%	57.40%	64.90%	71.28%
Type II (0/1)	29.00%	26.20%	25.70%	26.07%
Type III (1/0)	4.20%	5.80%	3.20%	2.05%
Type IV (0/0)	8.80%	10.60%	6.20%	0.00%
Observed Instances	278^∗^60 = 16680	308^∗^30 = 9240	222^∗^30 = 6660	195^∗^30=5850


## Discussion

Although there is currently much speculation about the potential adaptive significance of natural epigenetic variation, two key questions remain difficult to tackle: the temporal stability of natural epigenetic variation, and its degree of independence from genetic variation. Here, we addressed these questions using the clonal plant invader alligator weed as a model system. We show that invasive alligator weed populations harbor substantial epigenetic but very little genetic variation, so most epigenetic variation is independent. We also show that much of the epigenetic variation is maintained in a common environment and only gradually decreases over multiple generations. This transient epigenetic stability could play a role in environmental adaptation and invasion success.

### Genetic and Epigenetic Diversity in the Field

In many plant species, genetic and epigenetic variation co-occur in natural populations and are difficult to disentangle (Bossdorf et al., 2008). Here, we avoided this problem by working with an asexual species. Our MSAP analysis demonstrated abundant epigenetic diversity within and among invasive populations of alligator weed, and much of this epigenetic variation was independent of DNA sequence (“pure epigenetic variation” *sensu* Richards, 2008) as most of the samples (73.3%) shared the same AFLP multi-locus genotype. Similar contrasting levels of genetic vs. epigenetic variation have been reported in other asexual species, including the plants *Fallopia japonica* (Richards et al., 2012), *Pinus pinea* (Saéz-Laguna et al., 2014) and *Taraxacum officinale* (Preite et al., 2015), and the asexually reproducing fish *Chrosomus eos-neogaeus* (Massicotte and Angers, 2012). The high levels of epigenetic variation in genetically depauperate asexual species could have at least two reasons: First, epigenetic variation might be generally larger because of higher spontaneous epimutation rates than genetic mutation rates (van der Graaf et al., 2015), which may uncouple genetic and epigenetic variation. Second, the variation created by spontaneous epimutation can be transmitted and thus accumulated much more easily in asexual species where epigenetic reprogramming (i.e., the resetting of epigenetic modification) during gametogenesis and early embryo development is often circumvented through vegetative reproduction (Verhoeven and Preite, 2014).

### Temporal Stability of Epigenetic Variation

We found considerable epigenetic diversity within and among natural populations, which is consistent with previous studies (Kilvitis et al., 2014). However, virtually all previous studies sampled plants at only one point in time, producing a single snapshot of epigenetic dynamics (Lira-Medeiros et al., 2010; Medrano et al., 2014; Schulz et al., 2014; Foust et al., 2016). Still, epigenetic variation is at least partly sensitive to environmental change, and it is therefore generally difficult in such studies to assess the stability and representativeness of observed epigenetic patterns (Verhoeven and Preite, 2014). In our study, we used a repeated sampling strategy also for the field, and we found that the epigenetic diversity within and differentiation among natural populations were largely stable across multiple years, as indicated by the similar Shannon indices and the similar population positions in PCoA space. The differentiation among natural populations was not simply a result of accumulating random epimutations and isolation-by-distance, as some population pairs were epigenetically more similar than others, despite their larger geographic distance.

Most importantly, we found that epigenetic differences among populations were also maintained at first in a common environment, but then progressively eroded over multiple generations. This suggests that a large part of the epigenetic variation observed in the field was environmentally induced, but it did not behave like classic phenotypic plasticity which disappeared quickly (Geng et al., 2007) but instead showed greater inertia and was transiently stable for at least several asexual generations. These results were also confirmed by our locus-by-locus analysis of stability, where the stability of epiloci was a decreasing function of the duration of the experiment. Interestingly, we found some plastic epiloci to be statistically associated with climate variables (unpublished data), indicating that some of the meta-stable epigenetic differences may have been induced by climatic variation. However, the design of our study did not really allow to address the causal drivers of epigenetic population differences, and more research is needed to understand this.

Some epigenetic population differentiation remained significant even after 10 generations of cultivation in a common environment, suggesting that part of the differentiated epiloci were either only very slowly responding to a changing environment, or they might have been permanent, reflecting stably transmitted epigenetic variation, possibly resulting from epimutation and subsequent selection. Altogether, the epigenetic variation observed among invasive alligator weed populations appears to be combination of stable and environmentally induced variation, with the majority of the environmentally induced component showing a transient stability.

It is important to note that the MSAP data in our study was based only on DNA methylation in a CG context, but not in CHH or CHG contexts, and therefore our conclusions only apply to CG context. To understand the dynamics of other DNA methylation contexts, more powerfully, NGS-based methylation analyses are needed, and should be employed in future analyses.

### Implication for the Invasiveness of Alien Species

Asexual reproduction is often thought to be beneficial for the establishment and spread of alien plant species, because it provides reproductive assurance at invasion fronts where population densities are often low (Silvertown, 2008). However, the downside of such asexual spread is that the populations are often characterized by extremely low levels of genetic diversity, which may limit their adaptive potential in novel and heterogeneous habitats (Barton and Charlesworth, 1998). Epigenetic processes have been proposed to resolve this ‘genetic paradox’ of successful invasive species (Hawes et al., 2018): if epigenetic variation is more dynamic and rapidly generated in asexual populations, and it also associated with heritable phenotypic variation, this may significantly alleviate the evolutionary constraints in genetically depauperate invasive populations. In this case, epigenetic variation will have important effects on the invasiveness of alien species. Our results with alligator weed support this hypothesis. Similar results have been found for other invasive species (see Hawes et al., 2018 for a recent review). For example, Richards et al. (2012) found that the invasion of diverse habitats by invasive Japanese knotweed, another prominent clonal plant invader, is more correlated with epigenetic variation than with genetic variation. In a study of an introduced bird, Liebl et al. (2013) found that in invasive populations of house sparrows, genetic diversity decreased because of inbreeding but at the same time epigenetic diversity significantly increased. Thus, epigenetic variation may compensate for the loss of genetic variation in invasive species and a thus contribute to their success in novel environments.

## Author Contributions

YG, JY, and C-YX designed the research. WS, XC, and LG performed the wet lab work. YG performed the data analysis. WS and C-YX participated in the sampling. YG, JY, XO, OB, and C-YX drafted and revised the manuscript. All authors approved the final manuscript.

## Conflict of Interest Statement

The authors declare that the research was conducted in the absence of any commercial or financial relationships that could be construed as a potential conflict of interest.
