# Supplementary material for: Transient Stability of Epigenetic Population Differentiation in a Clonal Invader
Source: Front Plant Sci. 2019 Mar 1;9:1851. doi: 10.3389/fpls.2018.01851 (PMC6405520; doi:10.3389/fpls.2018.01851)
Supplement: Supplementary file 1 [file Table_1.docx]

**TABLE S1.** Epigenetic diversity of six populations of *Alternanthera philoxeroides* with three types of sub-loci.

| Dataset\Population | GL | JN | KM | NC | NN | WH | Pop-mean |
| --- | --- | --- | --- | --- | --- | --- | --- |
| a) Percentage of polymorphic loci (%) for different sub-loci | | | | | | | |
| MSAP u-type sub-loci (253) |  |  |  |  |  |  |  |
| Field 2012 | 11.46 | 3.16 | 8.3 | 8.7 | 9.88 | 6.72 | 8.04 |
| Garden 2013 | 10.67 | 1.19 | 6.72 | 7.51 | 10.67 | 10.67 | 7.91 |
| Garden 2014 | 9.49 | 1.98 | 7.91 | 9.88 | 9.88 | 8.3 | 7.91 |
| Field 2014 | 11.46 | 7.51 | 8.7 | 9.88 | 15.02 | 6.32 | 9.82 |
| Chamber 2016 | 1.39 | 1.05 | 1.39 | 1.39 | 3.83 | 1.39 | 1.74 |
| MSAP m-type sub-loci (258) |  |  |  |  |  |  |  |
| Field 2012 | 10.47 | 3.49 | 8.14 | 7.75 | 9.69 | 6.2 | 7.62 |
| Garden 2013 | 10.08 | 1.94 | 6.59 | 6.59 | 10.85 | 9.69 | 7.62 |
| Garden 2014 | 9.69 | 3.49 | 9.3 | 10.08 | 9.69 | 8.53 | 8.46 |
| Field 2014 | 10.85 | 8.14 | 8.91 | 10.08 | 12.02 | 5.43 | 9.24 |
| Chamber 2016 | 1.06 | 1.06 | 1.41 | 1.06 | 3.53 | 1.06 | 1.53 |
| MSAP h-type sub-loci (140) |  |  |  |  |  |  |  |
| Field 2012 | 8.57 | 1.43 | 2.86 | 7.86 | 5 | 2.14 | 4.64 |
| Garden 2013 | 8.57 | 2.14 | 1.43 | 5.71 | 3.57 | 4.29 | 4.29 |
| Garden 2014 | 4.29 | 0.71 | 5 | 2.86 | 3.57 | 2.86 | 3.22 |
| Field 2014 | 4.29 | 1.43 | 6.43 | 2.86 | 7.86 | 3.57 | 4.41 |
| Chamber 2016 | 1.23 | 0.62 | 0.62 | 0.62 | 2.47 | 1.85 | 1.24 |
| b) Shannon’s information index (I) for different sub-loci | | | | | | | |
| MSAP u-type sub-loci (258) |  |  |  |  |  |  |  |
| Field 2012 | 0.082 | 0.021 | 0.055 | 0.055 | 0.073 | 0.048 | 0.056 |
| Garden 2013 | 0.075 | 0.008 | 0.049 | 0.049 | 0.075 | 0.073 | 0.055 |
| Garden 2014 | 0.079 | 0.014 | 0.067 | 0.08 | 0.084 | 0.068 | 0.065 |
| Field 2014 | 0.075 | 0.047 | 0.055 | 0.056 | 0.11 | 0.044 | 0.065 |
| Chamber 2016 | 0.013 | 0.008 | 0.011 | 0.013 | 0.032 | 0.011 | 0.015 |
| MSAP m-type sub-loci (253) |  |  |  |  |  |  |  |
| Field 2012 | 0.076 | 0.022 | 0.056 | 0.05 | 0.072 | 0.045 | 0.054 |
| Garden 2013 | 0.072 | 0.013 | 0.047 | 0.045 | 0.075 | 0.067 | 0.053 |
| Garden 2014 | 0.081 | 0.028 | 0.078 | 0.081 | 0.084 | 0.071 | 0.071 |
| Field 2014 | 0.069 | 0.053 | 0.057 | 0.059 | 0.089 | 0.038 | 0.061 |
| Chamber 2016 | 0.01 | 0.009 | 0.011 | 0.009 | 0.03 | 0.008 | 0.013 |
| MSAP h-type sub-loci (140) |  |  |  |  |  |  |  |
| Field 2012 | 0.059 | 0.009 | 0.018 | 0.052 | 0.031 | 0.01 | 0.03 |
| Garden 2013 | 0.065 | 0.01 | 0.01 | 0.033 | 0.023 | 0.031 | 0.029 |
| Garden 2014 | 0.036 | 0.007 | 0.041 | 0.022 | 0.029 | 0.024 | 0.027 |
| Field 2014 | 0.028 | 0.007 | 0.039 | 0.013 | 0.05 | 0.023 | 0.027 |
| Chamber 2016 | 0.01 | 0.005 | 0.006 | 0.006 | 0.018 | 0.016 | 0.01 |
